# Young women smokers’ response to using plain cigarette packaging: qualitative findings from a naturalistic study

**DOI:** 10.1186/1471-2458-14-812

**Published:** 2014-08-07

**Authors:** Crawford Moodie, Linda Bauld, Allison Ford, Anne Marie Mackintosh

**Affiliations:** Centre for Tobacco Control Research, Institute for Social Marketing, University of Stirling, Stirling, FK9 4LA Scotland, UK; UK Centre for Tobacco and Alcohol Studies, Stirling, UK

**Keywords:** Tobacco, Plain packaging, Interviews, Naturalistic, Public health

## Abstract

**Background:**

The aim of this study was to explore in-depth the response of young women smokers (18–35 years) to using dark brown ‘plain’ cigarette packs in naturalistic settings.

**Methods:**

Participants were recruited in six towns and cities in Scotland to take part in a naturalistic study, where they used plain cigarette packs for a week. Participants completed a number of questionnaires during the study period (reported elsewhere), and a sub-sample participated in post-study telephone interviews to explore their experiences of using the plain packs. Of the 187 participants who completed the study, 23 were randomly selected to participate in the post-study interviews. Within the interviews a semi-structured topic guide was used to assess perceptions of the plain pack, feelings created by the pack, feelings about smoking, and avoidant and smoking behaviour.

**Results:**

The brown (plain) packs were perceived negatively due to the colour, the undesirable image the pack conveyed, and the reaction from others. The plain packs were also associated with negative feelings, such as embarrassment, discomfort and guilt. Some participants also commented that they felt differently about the product, considered to be less enjoyable or more harmful, when using the plain packs, and were less interested in, or felt more negatively about, smoking. A number of participants said that they had engaged in avoidant behavior with the plain packs, such as hiding it, due to their negative thoughts about the packs and the reaction of others. Some participants also mentioned cessation-related behaviours when using the plain packs, such as forgoing cigarettes, stubbing cigarettes out early and thinking about quitting, largely due to the decreased enjoyment of smoking.

**Conclusions:**

The experience of using cigarettes in plain packs prompted a range of negative responses from young women smokers, who are a crucial target group for tobacco control interventions.

## Background

In December 2012 Australia became the first country to require plain packaging for all tobacco products, with the governments of New Zealand, Scotland and the Republic of Ireland all announcing plans in 2013 to follow Australia’s lead. Plain (or standardised) packaging has also been proposed in other countries, such as India and the United Arab Emirates. This recent policy interest coincides with the growing body of evidence supporting the potential public health benefits of plain packaging. A systematic review of plain packaging research, commissioned by the UK government in 2011, included 37 studies [1], with a further 17 published studies identified within an update of the literature in 2013 [2]. As of March 2014, at least eight additional studies have been published [3–10]. The vast majority of these studies (53 of 62) have been conducted since 2007.

This growing body of evidence collectively suggests that plain packaging will: reduce pack appeal; increase the effectiveness of the on-pack health warnings, and; prevent colour and pack shape being used by consumers as an indicator of product harm. With respect to research from Australia since plain packaging was introduced, few published studies have assessed the response of smokers to the change in packaging. One such study, conducted in November 2012 when both plain and branded packs were on sale, found that smokers using plain packs, compared with smokers using branded packs, perceived their cigarettes to be less satisfying and poorer quality and were more likely to think about and prioritise quitting [11]. Another study found an increase in the number of calls to the Quitline in two Australian states (New South Wales and the Australian Capital Territory) following the introduction of plain packaging [12].

These findings from Australia are consistent with the quantitative findings from three naturalistic studies in Scotland and France, where young adult smokers were given brown ‘plain’ packs and instructed to use these for between seven and fourteen days [13–15]. Smokers in Scotland were asked to transfer cigarettes from their own fully branded packs into brown packs provided to them and use these for a period of one or two weeks [13, 14]. In France, smokers were asked to buy a ten-day supply of rolling tobacco, which was then transferred by market recruiters into plain roll-your-own packs, and use these plain packs for ten days [15]. In all three studies, smokers were asked to complete a number of questionnaires (developed from focus group research and the tobacco, plain packaging and marketing literature) during the study to allow for comparison between the plain and fully branded packs. Based on the responses to these questionnaires, plain packs were consistently associated with more negative perceptions of the pack (e.g. lower quality, less attractive), more negative feelings of using the pack (e.g. more embarrassed), more negative feelings about smoking (e.g. less enjoyable, less satisfying), avoidant behavior (e.g. hiding the pack) and cessation-related behaviours (e.g. reducing consumption, thinking about quitting).

Each naturalistic study also included post-study interviews with a random sample of participants to gauge their experiences of using the plain packs. However, no in-depth qualitative analysis has been reported for any of these studies [13–15]. To fill this important gap in the literature, our objective was to explore how young women smokers respond to the use of plain packaging, and the reasons underlying these responses, by analysing the post-study interviews for one of the naturalistic studies conducted in Scotland [14]. Young women were selected given their high smoking prevalence in the UK, and because packaging aesthetics have been found to be more important for young women than for young men [13, 16, 17].

## Methods

### Study design

Between June 2011 and March 2012 young women smokers (N = 301) were recruited, by market research recruiters, from eight postcode sectors from within the six most populated towns and cities in Scotland, using random location quota sampling. Postcode sectors were randomly selected and stratified by DEPCAT score, a measure of multiple deprivation, to ensure coverage of a range of socioeconomic backgrounds. Within each postcode sector, market recruiters were instructed to recruit participants using the door knock method. To satisfy inclusion criteria women had to be daily smokers between the ages of 18 to 35 years - a breath sample was used to confirm that they were smokers. Participants were informed that the study was concerned with smokers’ opinions of cigarette packaging and that participation would involve using cigarette packs provided to them and completing questionnaires twice a week. Those providing written consent were given a ‘completion’ pack containing brown packs with a fictitious brand name (Kerrods) to prevent copyright breach; questionnaires labelled by day and date; and a timetable explaining when to use the Kerrods packs and when to complete and return each questionnaire [14].

The study ran for two weeks, with the sample instructed to transfer cigarettes into plain packs and use these for one of the weeks. The Kerrods packs all had the text warning ‘Smoking kills’ on the pack front and one of three pictorial warnings on the back of the pack (an image of healthy and diseased lungs; an image of smoke in a child’s face; or a coloured text warning about seeking help). We provided all participants with a number of packs featuring different health warnings as smokers are accustomed to their cigarette packs displaying different warnings. The size, type and positioning of the warnings is consistent with how they appear in the UK. In the week following the main study, a sub-sample of participants from each of the six towns and cities were telephoned and invited to participate in a follow-up telephone interview exploring their thoughts and experiences of using the brown packs. We chose the week following completion of the main study to conduct the interviews so that the experience of using the brown packs would be fresh in participants’ minds. Telephone interviews were chosen ahead of face-to-face interviews for practical reasons, given that participants were recruited from towns and cities across Scotland.

### Sample

Of the 301 participants recruited, 187 (62.1%) completed all the questionnaires and reported using the correct pack when instructed to [14]. The sampling frame for the post-study interviews comprised these 187 participants. Participants were randomly selected, with stratification by location, to participate in the post-study telephone interview. Our aim was to conduct interviews with four participants from each of the six towns or cities. A total of 49 participants were contacted by two academics working within the Institute for Social Marketing (University of Stirling), both of whom had previous experience of conducting telephone interviews. Participants had been informed, prior to study onset, that they may be contacted by telephone in the week following the study to participate in a telephone interview, for which they would receive a payment of ten pounds. For those consenting to take part, it was explained that the interviews would last approximately thirty minutes and were concerned with their experiences of using the brown packs. Participants were also informed that there were no right or wrong answers and assured of confidentiality and anonymity.

Twenty-four participants agreed to take part, although one participant who had arranged a time for an interview was subsequently unavailable. As such, interviews were successfully conducted with 23 participants; eight declined to take part (this includes the participant who had originally agreed to participate but then dropped out) and the remaining 18 could not be reached by telephone after five attempts. For those who declined to participate, five said they were too busy, two mentioned personal or family problems, and one was not interested. Of the 23 participants who took part in the post-study interviews, 17 were aged 25–35 years and six aged 18–24 years, 15 from social grade ABC1 and eight from social grade C2DE; social grade was classified in accordance with the six groups used by the British National Readership Survey (A, upper middle class; B, middle class; C1, lower middle class; C2, skilled working class; D, working class; E, those at the lowest level of subsistence).

### Procedure

A semi-structured topic guide was developed by the research team to explore in more depth some of the issues that had been covered in the study questionnaires. The intention was to collect more detailed data regarding pack perceptions, feelings about the packaging, feelings about smoking, and avoidant and smoking behaviour. As an example, participants were asked how they felt when using the brown packs. These questions gave participants the opportunity to express in their own words their cognitive, emotional and behavioural responses to using the plain packs. Interviewers also probed for more detail based on how participants responded and adjusted the discussion in the interview to cover additional issues that the participant wanted to raise. The result was a discussion focused on the key issues but still consistent with a semi-structured, in depth interview.

The average interview lasted approximately 20 minutes (M = 19.8 minutes, range 10–35 minutes). Interviews were tape recorded and transcribed and thematic analysis conducted. This involved two members of the research team reading through all the transcripts and then identifying first high level codes, using the topic guide as a starting point. Further reading then produced subcategories through an iterative method. These categories were then used to code the transcripts, with coding carried out independently by two members of the research team and discussion to resolve any differences in interpretation. Ethics approval was obtained from the ethics committee of the Marketing Department at the University of Stirling.

## Results

A number of key themes emerged from the interviews. These were grouped into three main categories: pack perceptions; feelings created by the pack, and feelings towards the product and smoking; and avoidant and smoking behaviour. While themes sometimes overlap, each is presented separately with quotes followed by participant’s age and social grade. While the meaning and interpretation of what is said in qualitative research is more important than the number responding in a particular way, some quantification can improve the transparency of data analysis, give precision to statements, enable patterns in the data to emerge with greater clarity and increase the meaning of key findings by providing focus [18]. For these reasons, and as all interviewees were asked exactly the same core questions, for each of the main themes we provide information on how many participants responded in a particular way to each question. It is important to note, however, that doing so does not allow for broader generalisation to be made beyond this study [18].

### Pack perceptions

Comments about how participants thought about the pack encompassed their general perceptions of the pack, views on the colour, and reports of how other people reacted to seeing the plain packs.

#### General pack perceptions

Most participants (16 out of 23) perceived the brown (plain) packs negatively, considering them to be ugly, lacking in style and strange, e.g. “I just didn’t think the packet was like stylish and I didn’t like the look of it basically” (18, C1). The brown pack was described as inconsistent with their image of the ‘type’ of cigarette that they, as young women, would use. For instance, participants mentioned that the packs were for older people, e.g. “My gran smokes fags similar, well the packet, the same sort of packet” (22, E), or the homeless, e.g. “It reminded me of tramps sitting in a park drinking with a brown paper bag, except [*this was*] smoking” (33, E).

*I don’t know why I kind of associate it with kind of like old men’s fags because I don’t know whenever I’ve just seen that colour of packet it’s always been kind of older men, and that’s not something that appeals to me* (25, C1)

The seven remaining participants felt the packets were no different, e.g. “It wasn’t any different from using my Benson & Hedges packet” (35, C1). For some, addiction was more important than the packaging, e.g. “As a smoker the important thing to me is actually the cigarettes, not what they come in, you know packaging is not, maybe I've been smoking for so long it doesn't matter what they come in because it's cigarettes that I am in need of, that sounds terrible, it’s addiction” (35, C2).

*It's an addiction. It's not about the packet, it's about the cigarette inside the packet* (35, C1)

#### Colour

Fourteen participants made negative comments about the brown pack colour, described as cheap, dull, dirty, unattractive and horrible. Seven participants were indifferent to the pack colour, while the two remaining participants liked it as it was unusual, or similar to a different brand: “I actually thought it was quite nice. It’s very similar to a Benson and Hedges pack” (34, C1).

*It was just like the colour of it and everything, it wasn’t very trendy, that’s the only thing I found really awkward about using it* (35, C1)

*Now the packets are more appealing. They’re nicer colours, I think it’s all to do with the colour, that brown colour I didn’t like at all, usually I’ve got ones that are like blue with a nice pattern on them and silver around it and stuff* (25, C1)

Five participants commented that the colour made them think about some of the dangers associated with smoking, for instance, by drawing a connection between the colour and respiratory disease, e.g. “Brown makes you think of what cigarettes do to your lungs” (26, B).

*I think the colour does make you think - you associate the colour of the packet with what your lungs would be like. It did enter my head* (26, B)

#### Reaction from others

Twenty-one participants said the pack drew attention from others, both smokers and non-smokers, in the form of direct questions or comments, with four of these participants also noticing other people staring at the pack or receiving “funny looks” from others (33, C1). Although not directly asked, nine participants reported feeling embarrassed, self-conscious or fed-up with the attention, e.g. “I felt self-conscious and felt people were generally looking at it, but out of curiosity” (21, E).

The most common questions participants were asked by others were what kind of cigarettes they were smoking, why they were using that pack and where the pack was from. Some participants felt the legitimacy of the product was questioned, e.g. “What is that you are using? Where did you get those cigarettes? I think it was along the lines of they look like fake or illegal cigarettes” (26, B). Several also felt the packs were the subject of amusement, e.g. “A couple of my friends were kind of laughing about it” (25, C1).

Two participants reported situations where friends or a boyfriend had refused to take a cigarette from the plain pack, e.g. “My boyfriend, he wouldn’t take any fags out of my packet” (30, C1). Six participants said that other people had commented that the pack would be a useful tool to help people realise the harms of smoking or act as a deterrent.

*My sister came in that day… she was like, what are they? I went it’s my normal fags, it’s a different packet. She was, I wouldn’t walk about with them* (30, C1)

*They* [friends] *said that it would put you off buying them if they were like that* (25, C1)

The two participants who said they had no reaction from others explained that this was because they did not take the pack out in front of other people: “I kept the packs well hidden” (35, C1).

### Feelings created by the packs, and feelings about the product and smoking

Participants described their emotional response to using the plain pack, in particular the feelings created by the packaging, feelings about the product, and how the pack made them feel about smoking.

#### Feelings created by the packaging

Eighteen participants commented that they felt differently when using the brown pack, for instance, embarrassed, self-conscious, uncomfortable, ashamed or guilty, e.g. “It did definitely make me feel guilty, I would say, which I don’t normally do with my own pack” (26, B). Another participant commented that the pack made her feel dirty: “The funny thing is you feel more dirty” (35, C1). Five of these participants said that although they initially felt differently, for instance embarrassed or apprehensive, they quickly overcame such feelings after using the pack for a day or two, e.g. “The first few days I felt strange because it was a brown pack and not a green pack, just obviously because I smoke menthol, but after a few days I got used to it” (33, C1). The five remaining participants reported feeling no differently when using the pack: “To me a packet is a packet” (25, E).

*Sometimes I felt like ashamed pulling out the brown packet because it was all dark and dull and it wasn’t like, see, the same as the normal packet* (18, C1)

*I didn’t like it. I just wasn’t comfortable* (35, C1)

#### Feelings about the product

Although not directly asked about their feelings about the product, eight participants specifically made comments concerning how they felt about cigarettes from the plain pack. The comments made typically related to enjoyment and/or harm. The plain pack was felt to reduce enjoyment of the cigarette, e.g. “I wasn’t enjoying the cigarette as much when I had that packet” (29, E), with this lack of enjoyment sometimes the consequence of increased feelings of guilt when smoking: “I was feeling guilty when I was smoking it and then not enjoying it” (21, E).

*In general, I just didn’t enjoy it as much – the whole experience of having a normal cigarette like I normally do* (26, B)

The plain pack was also felt to increase awareness of the harms associated with smoking, e.g. “The initial thought was the damage I’m doing to myself” (29, E). One participant reported feeling confused with the plain pack and had to reassure herself that it was her own brand of cigarette she was smoking, e.g. “A few times I forgot all about it and I had to look at the fag again to see it was my fag” (22, E).

*I didn’t feel like I normally do when I have a cigarette… It’s the thought of what it does to your lungs and the whole health risk around it* (26, B)

#### Feelings about smoking

Eight participants said that they felt differently about smoking when using the brown pack. For instance, smoking was considered less enjoyable, or more embarrassing, e.g. “I think because the packet made me feel slightly embarrassed it then led me on to feel a bit more embarrassed about smoking as a whole” (33, E). Several participants specifically stated that the packs made them feel like they were ‘doing something wrong’, e.g. “It kind of made me feel more like I was doing something wrong, I don’t know why it’s just because they stood out more, it did honestly make me feel worse about smoking than I normally do” (26, D). Comments were also made about reduced interest in smoking with the plain pack: “When it comes to smoking, it made me not really want to smoke as much. It made me feel a wee bit less interested in smoking” (22, E).

*I felt like I was doing something wrong with the brown packet, I know I’m doing something wrong anyway, but I felt more highlighted to it with the brown packet* (33, E)

*I know it’s in different coloured packets but every time when I’ve got my normal packet I want to smoke more, but when I’ve got that brown pack I’m not bothered because it is in a different packet, it’s like a common packet* (18, C1)

### Avoidant and smoking behaviour

Participants were asked whether they concealed the plain pack, and about their smoking behaviour while using the plain pack.

#### Avoidant behaviour

Thirteen participants indicated that they had hidden the plain pack, leaving it in their pockets or bags when they removed a cigarette, e.g. “I was hiding the cigarette pack and taking out a cigarette without anybody seeing the pack” (35, C1). For one participant this only happened for the first day. The most prominent reason offered for hiding the pack was its lack of appeal, being considered boring, unattractive, off-putting or disgusting, e.g. “I just kept well hidden in my pocket or in the jacket or in my bag, no I wouldn’t use them around people… I think it was just giving out the message that it was disgusting and, you know, a message was coming across in my head that it’s not nice to smoke” (35, C1). Others hid the pack, mostly from strangers, to avoid unwanted attention from others, or a feeling of shame: “I did leave it in my bag a lot. When I needed a cigarette I just took cigarettes out. So it obviously did, there was obviously part of me that didn’t really want to, you know, bring it out or have people ask me what it was about” (26, B). The remaining ten participants did not indicate that they had concealed the pack.

*Usually I pull my cigarette packet out no problem, but I did feel I had to hide it… I’m ashamed of smoking as it is. Then it was just like more attention was brought to me because I had a packet that was different* (29, E)

*I rarely took them out of my bag cause I did think they looked pretty horrible… I just think it is more, probably is more of an image thing, more than anything else. I don’t think they were awful, but it is obviously that they aren’t that nice to look at really* (21, E)

#### Missing cigarettes or stubbing them out early

Five participants reported missing out cigarettes, offering a variety of reasons. For some, this was due to reduced enjoyment, e.g. “On some nights maybe I would have cut down, maybe about three or four, which is good for me. You know as the week went on and I thought, I could definitely say I smoked a wee bit less… I wasn’t enjoying it the same, because of the pack” (35, C1). For others however, the packaging appeared to act at a subconscious level as they had not initially attributed missing out cigarettes to the plain pack, e.g. “I did miss out on some cigarettes and at the time I didn’t realise that it was the packaging but I think maybe it was because I’ve been smoking slightly more now that I am using my own brand packet” (33, E). Other reasons for missing out cigarettes included feeling less attached to the pack, as if it was not their own, and also the pictorial health warnings and completing the survey. No participant mentioned smoking more when using the plain packs.

*There was a couple of times I didn’t have one because of it and it did make me think about, like, giving up smoking… I think it was to do with the packet though, like, I probably wouldn’t go into a shop and want to buy a packet like that* (25, C1)

*I smoked less with the brown packet than what I usually do… Just cause I didn’t feel like it was my fag packet cause it was a different colour* (18, C1)

Four participants indicated that they had not forgone any cigarettes but did stub cigarettes out early as a consequence of the plain packs, e.g. “I don’t really feel it affected the amount I had, but it would probably affect how long I smoked a cigarette for and whether I would smoke a cigarette or stub it out early” (21, E). The most common reason for stubbing cigarettes out was because they were considered to be less enjoyable, although interestingly one participant also felt the cigarette was too strong: “I wasn’t enjoying the cigarette as much when I had that packet… I’d light a cigarette and I couldn’t smoke it, it was too strong, then I’d put it out. That is something I’ve never ever done in my life and I’ve smoked for a long, long time” (29, E).

*Just felt sort of less enjoyable, like I didn’t want to have it, I’d put it out earlier than what I would normally* (33, C1)

#### Thoughts of quitting

Although not asked specifically about quitting, three participants who had reported missing out on cigarettes also mentioned that the plain pack had made them think about quitting, or reinforced their desire to do so, e.g. “I want to stop and when I was taking out of the brown packet I was saying to myself should I be smoking this or should I be stopping, but it is hard to stop” (18, C1). One participant, who had not reduced consumption or stubbed out cigarettes early, also indicated an increased desire to quit: “It definitely did make me think about stopping smoking a lot more” (26, D).

## Discussion

Although plain packaging was fully implemented in Australia in December 2012 there is, at time of writing, no published qualitative research exploring how smokers have responded to this change of packaging. There also remains a dearth of naturalistic research in countries that do not have plain packaging, even though a strength of this approach is that it allows an insight into how, and more importantly why, smokers react the way they do to plain packaging. In this study, following the use of brown plain packs in naturalistic settings, young women smokers participated in a telephone interview and were asked to describe, in their own words, their cognitive, emotional and behavioural responses to using these packs.

For some young women, plain packs were perceived as having little impact and viewed as ‘just another pack’, a container for the product that they are addicted to. For many however the plain packs were thought of negatively, viewed as unappealing and inconsistent with their image of the ‘type’ of cigarette that they or other young women would use. The brown pack colour evoked associations of older men, elderly relatives and tramps, which is far removed from the user image that tobacco companies aim to create when investing in female-oriented brands [19, 20]. The findings highlight the importance of base colour for plain packaging. For marketers colour is often viewed as the most influential aspect of packaging design [21, 22], and aside from reducing pack appeal, the brown pack colour also highlighted some of the harms that can be caused by smoking, such as lung damage. The fact that dark brown, and darker colours in general, are viewed as signaling greater product harm is consistent with the plain packaging literature [1, 2] and tobacco industry marketing documents [23].

While most participants disliked the brown packaging, for many it also generated negative emotions, such as guilt, shame, embarrassment and discomfort. It also influenced how they felt about the product and about smoking. Several participants described the cigarette as less enjoyable from the plain pack and as something that created guilt or greater perceptions of harm. Transferring feelings about the pack to the product, or ‘sensation transfer’, has long been recognised in the marketing literature [24–29] and in tobacco industry documents [30, 31]. For instance, Philip Morris research found that most smokers appraised cigarettes that were identical in composition as “too mild” from a blue pack while others felt they were “too strong” from a red pack. This study echoes our findings, with one young woman mentioning that her cigarettes from her usual fully branded pack tasted ‘too strong’ in the plain pack, even though it was the same cigarettes in both packs. For others, the plain pack also influenced feelings about smoking, reducing both enjoyment and interest in smoking while increasing embarrassment and the feeling that they were ‘doing something wrong’.

For some participants the reduced appeal of the plain pack and the negative reactions of others provoked avoidant behaviour, such as hiding the pack. The plain pack also appeared to encourage cessation related behaviour, with a number of participants mentioning forgoing cigarettes, stubbing them out earlier or thinking about quitting when using the plain pack. Reduced enjoyment from smoking was the most prominent factor underlying these actions. However, the young woman who had mentioned her cigarettes were ‘too strong’ said that she stubbed out cigarettes for this reason, and another young woman who had not initially attributed reduced consumption to the plain pack commented that she smoked more when returning to her fully branded pack. That this participant reported smoking more when using fully branded packaging may reflect the ability of pack design to influence usage at a subconscious level, as suggested within the marketing literature [32]. These findings provide depth to the quantitative results presented in the naturalistic study [14] and a bit more understanding of the possible ways in which plain packaging may influence smoking behaviour.

The study has a number of limitations that may have had an impact on how participants responded, such as the fact that plain packs are not available in Scotland, and therefore novel, and also the short study duration. If all cigarette packs on the market had a uniform appearance, thereby eliminating any possible novelty effect, the reactions from both smokers and others may have been different. Similarly, for those smokers who had negative feelings about the packs, and those who reported altering their smoking behaviour as a consequence of using the packs (e.g. reduced consumption or stubbed cigarettes out early), we are unable to determine whether this would be sustained over the longer term. In addition, for smokers who reported a change in smoking behaviour, this could be a consequence of socially desirable responding due to study participation. Another potential limitation is the reliance on the self-reported use of the brown packs. The study also provides no insight into how younger or older male and female smokers respond to using plain packaging, although young women are a key target group for public health interventions. Nevertheless, further research exploring how and why younger and older male and female smokers respond to plain packaging would be fruitful.

## Conclusions

Tobacco companies’ own research reveals how identical cigarettes presented in packs with different colours and designs lead some consumers to experience and evaluate them differently when smoked [33]. This is consistent with our findings where, over a period of a week, a plain brown pack design changed the product perceptions of young women smokers and, for some, had a demonstrable impact on how they feel and think about smoking and their smoking behaviour.
